# Left ventricular remodeling after reperfused acute myocardial infarction: insights from automated ECV mapping

**DOI:** 10.1186/1532-429X-18-S1-Q67

**Published:** 2016-01-27

**Authors:** Heerajnarain Bulluck, Steven K White, Stefania Rosmini, Amna Abdel-Gadir, Anish N Bhuva, Thomas A Treibel, Marianna Fontana, Patricia Reant, Manish Ramlall, Ashraf Hamarneh, Alex Sirker, Anna S Herrey, Charlotte Manisty, Peter Kellman, James Moon, Derek J Hausenloy

**Affiliations:** 1 grid.83440.3b0000000121901201The Hatter Cardiovascular Institute, University College London, London, United Kingdom; 2 Cardiac Imaging, Barts Heart Centre, London, United Kingdom; 3 grid.94365.3d0000000122975165NIH, Bethesda, CA USA

## Background

In reperfused acute ST-segment elevation myocardial infarction (STEMI), infarct size and microvascular obstruction (MVO) are strong predictors of adverse LV remodeling and poor outcome. However, the role of remote myocardium is less clear. Remote native T1 changes have been linked with adverse LV remodeling and is thought to be associated with inflammation. We used automated ECV mapping to provide further insights into the pathophysiology of LV remodeling.

## Methods

40 STEMI patients underwent CMR imaging at 1.5T (Avanto, Siemens), 4 ± 2 days post primary percutaneous coronary intervention and at 5 ± 2 months. Left ventricular (LV) short-axis native and post-contrast T1 (MOLLI) and T2 maps were acquired, and automated ECV maps generated (motion-corrected and co-registered). Mean segmental T2, T1 and ECV values were obtained as per the AHA model (CVI42, Calgary, Canada) (Fiure 1). Apical slices were excluded to avoid partial voluming errors. LV remodeling was defined as 20% increases in LV end-diastolic volume on the follow-up scan. Salvaged myocardium was defined as regions of high T1/T2/ECV without LGE.

## Results

8/40(20%) patients had AR at follow-up.

## Remote myocardium

In patients with (compared to without) LV remodeling, at baseline, native T1 and ECV values were higher in remote myocardium (T1: 1072 ± 54 ms vs. 1023 ± 46 ms;P = 0.01: ECV: 29.5 ± 1.4% vs. 27.3 ± 2.2%; P = 0.01). T2 values in the remote myocardium were identical (51.5 ± 2.6 ms vs. 49.4 ± 2.9 ms, P = 0.08).

## Acute infarct ECV

In patients with (compared to without) LV remodeling, at baseline there were larger infarct size and higher ECV compared to those without (LGE: 40 ± 8% vs 20 ± 11%, p < 0.001; ECV: 74 ± 8% versus 68 ± 10%, P = 0.03). However there was no difference in ECV, T1 and T2 of the salvage myocardium and T1 and T2 of the infarct zone.

## ECV and segmental recovery

153/480 segments had late gadolinium enhancement and abnormal wall motion. Mean segmental ECV performed as well as transmural extent of infarct to predict improvement in segmental wall motion with AUC 0.70 (95%CI 0.615-0.77) versus 0.72 (95%CI 0.64-0.79), P = 0.58. Binary logistic regression showed ECV to be an independent predictor of wall motion recovery after adjusting for LGE, T1 and T2 (odd ratio 0.941, 95% CI 0.886 - 0.999, P = 0.046).

Multiparametric prediction of remodelling.

Although infarct size was the strongest predictor, on multivariable regression, the remote myocardial ECV and then the presence of haemorrhage (low infarct core T1) was most associated with remodeling (Table 1).

**Table 1:** Result of univariable and multivariable regression analysis of factors associated with percentage increase in EDV

	Univariable Analysis			Multivariable Analysis	
	Regression slope	R2	P	Regression slope	P
ECV remote	3.27	0.24	0.001	2.8	0.007
Remote T1	0.13	0.20	0.004		
T1 MVO	-0.60	0.14	0.02	-0.05	0.039
EF	-0.64	0.13	0.03		
Acute infarct ECV	0.53	0.11	0.04		
baseline EDV	0.02	0.003	0.76		

## Conclusions

Not all infarcts are the same. The ECV of an infarct is a major predictor of regional functional recovery, whilst this ECV - but more strongly, the remote ECV predicts adverse remodeling.

**Figure 1:**
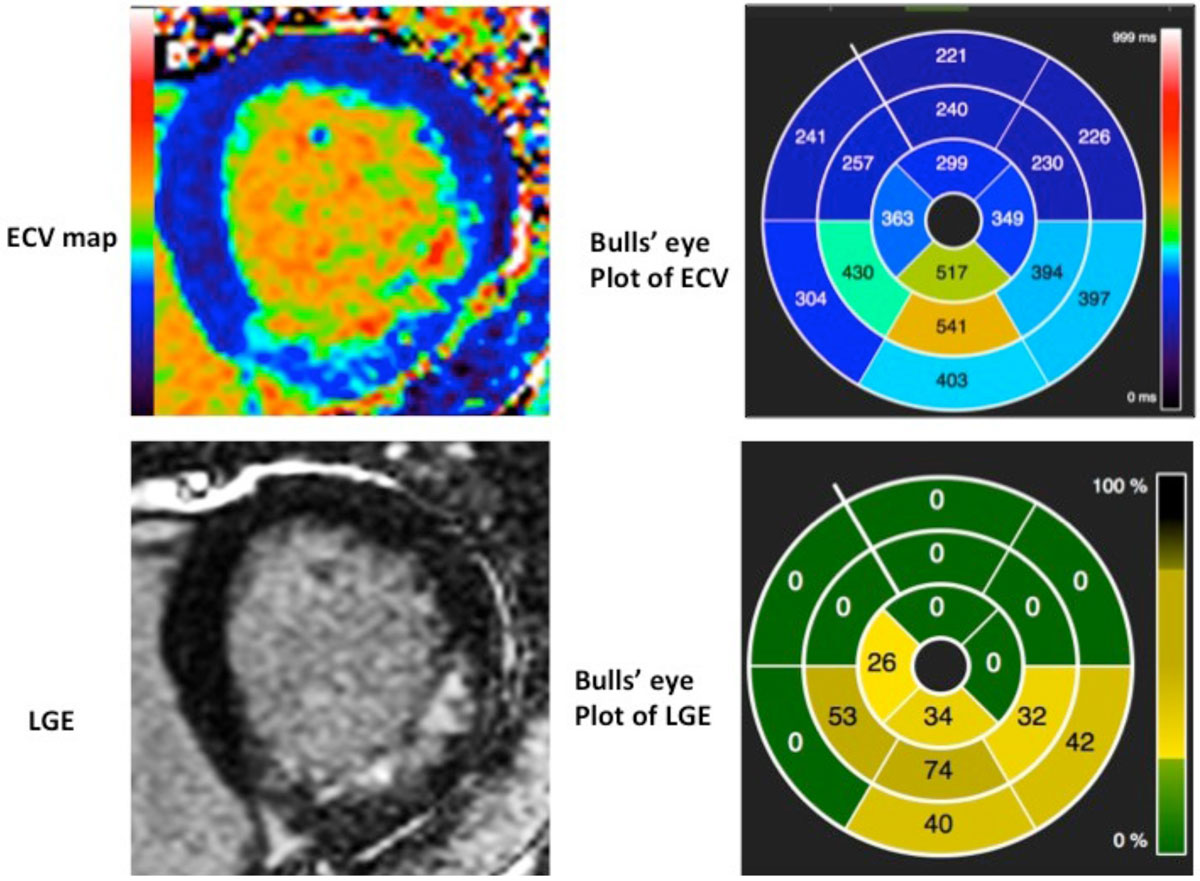
**Example of an automated ECV map and corresponding LGE image and bulls' eye plots**.

